# mTOR acts as a pivotal signaling hub for neural crest cells during craniofacial development

**DOI:** 10.1371/journal.pgen.1007491

**Published:** 2018-07-05

**Authors:** Xuguang Nie, Jinxuan Zheng, Christopher L. Ricupero, Ling He, Kai Jiao, Jeremy J. Mao

**Affiliations:** 1 Center for Craniofacial Regeneration, College of Dental Medicine, Columbia University, New York, New York, United States of America; 2 University of Alabama at Birmingham, Department of Genetics, Birmingham, Alabama, United States of America; 3 Department of Pathology and Cell Biology, Columbia University, New York, New York, United States of America; Brigham and Women’s Hospital, UNITED STATES

## Abstract

mTOR is a highly conserved serine/threonine protein kinase that is critical for diverse cellular processes in both developmental and physiological settings. mTOR interacts with a set of molecules including Raptor and Rictor to form two distinct functional complexes, namely the mTORC1 and mTORC2. Here, we used novel genetic models to investigate functions of the mTOR pathway for cranial neural crest cells (NCCs), which are a temporary type of cells arising from the ectoderm layer and migrate to the pharyngeal arches participating craniofacial development. *mTOR* deletion elicited a proliferation deficit and excessive apoptosis of post-migratory NCCs, leading to growth arrest of the facial primordia along with midline orofacial clefts. Furthermore, NCC differentiation was impaired. Thus, NCC derivatives, such as skeletons, vasculatures and neural tissues were either rudimentary or malformed. We further demonstrate that disruption of *mTOR* caused P53 hyperactivity and cell cycle arrest in cranial NCCs, and lowering P53 activity by one copy reduction attenuated the severity of craniofacial phenotype in NCC-*mTOR* knockout mice. Remarkably, NCC-*Rptor* disruption caused a spectrum of defects mirroring that of the NCC-*mTOR* deletion, whereas NCC-*Rictor* disruption only caused a mild craniofacial phenotype compared to the *mTOR* and *Rptor* conditional knockout models. Altogether, our data demonstrate that mTOR functions mediated by mTORC1 are indispensable for multiple processes of NCC development including proliferation, survival, and differentiation during craniofacial morphogenesis and organogenesis, and P53 hyperactivity in part accounts for the defective craniofacial development in NCC-*mTOR* knockout mice.

## Author summary

mTOR is a highly conserved serine/threonine protein kinase that plays critical roles in diverse processes. mTOR acts through forming two distinct functional complexes, namely the mTORC1 and mTORC2. In this study, we generated novel genetic models to investigate the functions of the mTOR pathway for neural crest cell (NCC), which is an important embryonic cell type for craniofacial development. Disruption of *mTOR* elicited a proliferation deficit and excessive cell death of post-migratory NCCs, leading to facial growth deficiency and orofacial clefts. Furthermore, NCC differentiation was impaired and NCC derivatives, such as skeletons, vasculatures and neural tissues were either rudimentary or malformed. We also found that disruption of *mTOR* caused P53 hyperactivity, and lowering P53 activity ameliorated the craniofacial phenotype seen in NCC-*mTOR* knockout mice, suggesting that P53 hyperactivity might in part account for the craniofacial defects in mutant mice. Remarkably, disruption of mTORC1 by deletion of the gene encoding the core component Raptor caused a spectrum of defects mirroring that of the NCC-*mTOR* knockout mice, suggesting that mTOR regulates NCC development principally through the mTORC1 pathway during embryogenesis.

## Introduction

Initiation of craniofacial morphogenesis is marked by the appearance of the paired pharyngeal arches (PAs). The first pair of PAs are further divided into paired mandibular and maxillary prominences, which together with the single frontonasal prominence (FN) constitute the five facial primordia. The facial primordia expand rapidly and unify to form a continuous face. An important cell type in this process is the cranial neural crest cells (NCCs), which are stem-cell-like cells formed at the border of the non-neural ectoderm and the developing brain, including the hindbrain, midbrain and posterior portion of the forebrain [1–4]. Cranial NCCs, upon induction, delaminate from the dorsal edge of the developing brain, migrate bilaterally along delicately organized paths, colonize at the ventral portion of the brain, and drive the budding of the five pairs of PAs and the single FN in mammals [1–4].

Post-migratory NCCs are highly dynamic in spatial distribution in response to local developmental cues, and proliferate vigorously to promote the rapid expansion of PAs and derivation of the facial primordia. Meanwhile, NCCs interact with local epithelial cells and mesoderm-derived mesenchymal cells to initiate organogenesis in the majority of craniofacial organ systems. The majority of cranial NCCs eventually commit to a fate decision depending on a specific developmental context and differentiate into a variety of cell types, including chondroblasts, osteoblasts, odontoblasts, melanocytes, vascular smooth muscle cells (SMCs), glial cells and neurons to form cartilages, bones, dentin, vascular smooth muscles and neural tissues [5]. Even though, a number of NCCs retain their stemness to maintain a stem cell pool in continuous development [3].

NCC development is finely controlled by a complex molecular network. Multiple lines of evidence indicate that the mammalian target of rapamycin (mTOR), which is a highly conserved serine/threonine protein kinase belonging to the phosphoinositide 3-kinase (PI3K)-related kinase family and integrates a myriad of signals for cell function, is potentially important for NCC development [6–10]. mTOR maintains tissue and organ homeostasis by regulating a variety of cellular processes, including proliferation, apoptosis, differentiation, metabolism, autophagy and aging. mTOR interacts with a set of molecules, including Raptor and Rictor, to form two distinct functional complexes, namely the mTORC1 and mTORC2 [7, 8, 11]. In physiological settings, mTORC1 senses diverse intracellular and extracellular cues, including signals of growth factors and amino acids, and regulates various cellular activities through phosphorylating eukaryotic translation initiation factor 4E (eIF4E) binding protein 1 (4E-BP1) and S6 kinase 1 (S6K1), which are critical for protein synthesis and ribosome biogenesis [6–9]. mTORC2 positively regulates mTORC1 through regulating Akt signaling [11–13].

Developmental roles of mTOR have been extensively studied in recent years. Germline disruption of *mTOR* in mice leads to embryonic death at the implantation stage [14]. Disruption of either *Rpto*r or *Rictor* also causes early embryonic death prior to craniofacial organogenesis, demonstrating that mTORC1 and mTORC2 are both critical for early embryonic development [12, 15]. Conditional knockout (cKO) approaches have unveiled critical functions of the mTOR pathway in a variety of organs including the brain, lung, heart, skin, muscle and skeleton [10, 16–20]. In addition, mTOR is also important for maintaining the stemness of various types of stem cells, and aberrant mTOR activities are implicated in diverse pathological conditions [7, 21, 22].

In the dental-craniofacial complex, functions of the mTOR pathway are less recognized. Recent studies indicate that mTOR is critical for craniofacial development. Disruption of *mTOR* in mesoderm-derived mesenchymal cells not only disrupts long bone development but also causes calvarial defects [10]. By contrast, hyperactivity of mTOR in NCCs caused by disruption of *TSC1*, whose product binds to TSC2 to suppress mTORC1 activity, leads to sclerosis in NCC-derived bones [23]. In spite of the potential significance of the mTOR pathway for NCCs, there still lacks direct evidence to assign a functional role for mTOR in NCC development. Here, we generated NCC-specific knockout models for the mTOR pathway using a widely used Cre strain containing the *Wnt1-cre* transgene, which specifically targets the majority of the pre-migratory NCCs if not all, and hence their descendants in the target organs [24]. We demonstrate that mTOR is indispensable for post-migratory NCC proliferation, survival and differentiation, and mTOR acts largely through mTORC1 during embryogenesis. Furthermore, mTOR deficiency induces P53 hyperactivity in NCCs.

## Results

### p-mTOR dynamics in cranial NCCs and their derivatives

**Fig 1:**
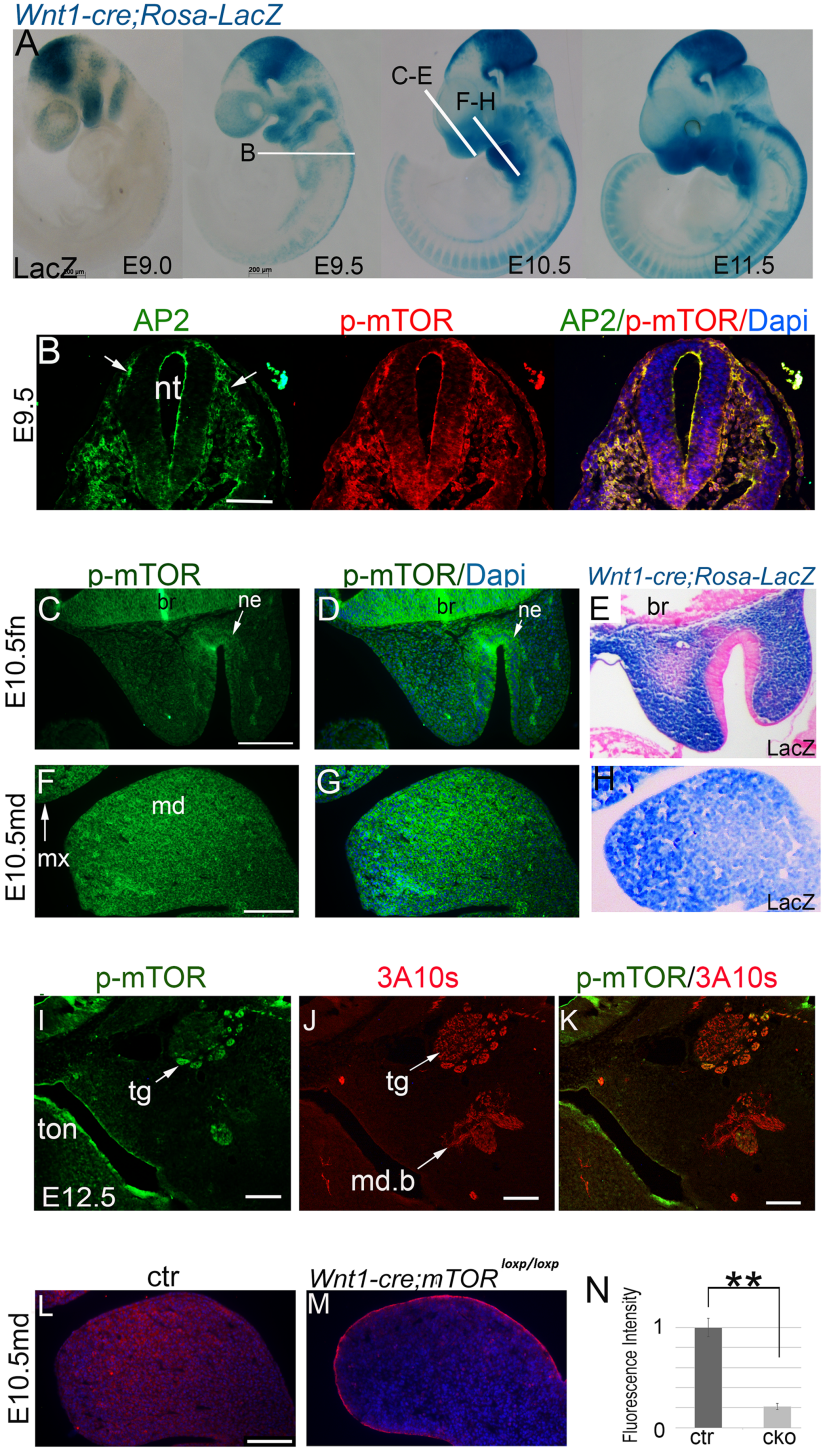
mTOR dynamics during craniofacial development. (A) Whole mount LacZ staining of *Wnt1-cre; Rosa26R* embryos. White lines indicate the sectioning planes in relevant images. (B) Immunofluorescence for Ap2-α and p-mTOR in the migrating NCCs at E9.0. (C, D; F, G) Immunofluorescence for p-mTOR in the FN and mandibular prominences at late E10.5. (E, H) Sections of a Lac-Z-stained E10.5 heads, showing NCC distribution in the FN and mandibular prominence. (I, J, K) Co-localization of p-mTOR with a neurofilament marker 3A10. (L, M) Immunofluorescence for p-mTOR in the mandibular prominence of E10.5 mice. (N) Quantification of p-mTOR levels of the mandibular prominences by calculating relative fluorescence intensity, **p<0.01. br: brain; ctr: control; fn: frontonasal prominence; cko: conditional knockout; md: mandibular prominence; md.b: mandibular branch of trigeminal nerve; mx: maxillary prominence; ne: nasal epithelium; nt: neural tube; tg: trigeminal ganglion; ton: tongue. Scale bar in (A): 200 μm; Scale bars in others: 100 μm.

NCC migration from the interface of the ectoderm and developing brain starts around E8.5 and completes during E9.5 to E10.5 in mice (Fig 1A). At E9.0, p-mTOR was observed in migrating NCCs at a high level, as indicated by co-localization of p-mTOR with a NCC marker AP2-α (Fig 1B). It also showed ubiquitous activation in the first pair of PAs, which are predominately composed of post-migratory NCCs (Fig 1A; S1 Fig)[25]. One day later, p-mTOR continued to maintain a high level in the expanding facial primordia, including the paired mandibular and maxillary prominences and the single FN, which are still largely populated by NCCs (Fig 1C–1H). During E11.5 to E12.5, when organogenesis has initiated in multiple systems, p-mTOR was found in mesenchymal condensates of the mandible and in neural tissues at high levels (S1 Fig; Fig 1I–1K). At mid-gestation, p-mTOR was found in NCC–derived bones and cartilages at medium levels (S1 Fig). Additionally, it was also seen in the craniofacial muscles, which are not derived from NCCs (S1 Fig). By contrast, p-mTOR levels in the first pair of PAs of the *Wnt1-cre;mTOR*^*loxp/loxp*^ cKO mice were markedly reduced, indicating that *mTOR* had been successfully disrupted by Cre-mediated recombination (Fig 1L, 1M and 1N).

### Disruption of *mTOR* causes defective craniofacial morphogenesis

**Fig 2:**
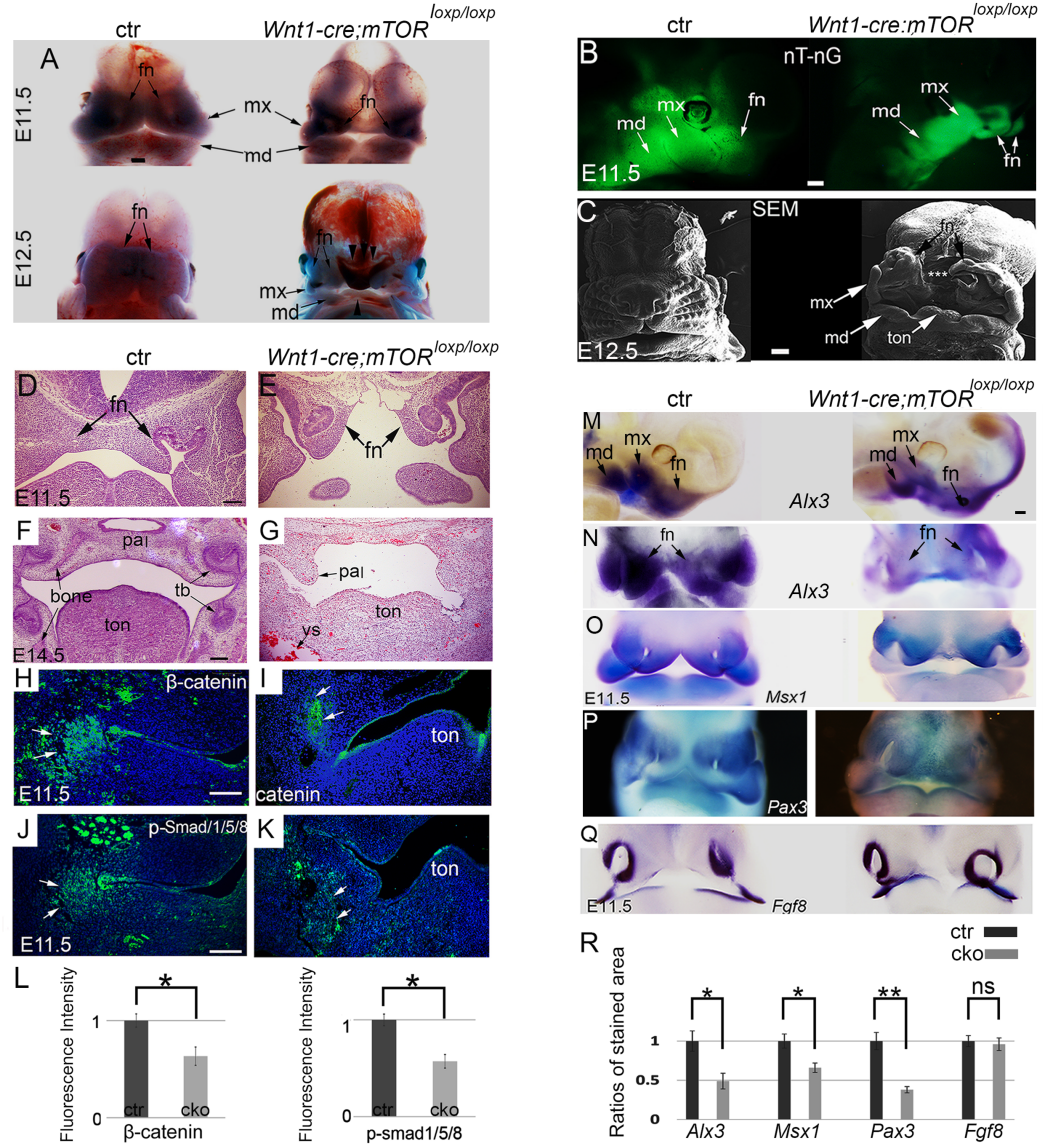
Defective craniofacial morphogenesis and organogenesis in the NCC-*mTOR* KO mice. (A) Craniofacial morphogenesis at two successive stages. Arrowheads point to facial cleft. (B) Lateral view of E11.5 heads, NCCs are GFP-labeled. (C) SEM examination of E12.5 heads, * marks facial cleft. (D, E) Histology of E11.5 heads, showing failed midline fusion of the mutant FNs. (F, G) Histology of E14.5 heads. (H-K) Immunofluorescence for β-catenin and p-Smad1/5/8, arrowheads point to positive staining in the mandibular prominences. (L) Quantification of β-catenin and p-Smad1/5/8 levels by calculating relative fluorescence intensity. (M-Q) Whole mount in situ hybridization for *Alx3*, *Msx1*, *Pax3* and *Fgf8*. (R) Quantification of in situ hybridization staining of *Alx3*, *Msx1*, *Pax3* and *Fgf8*. *P<0.05; ** P<0.01. br: brain; ctr: control; fn: frontonasal prominence; md: mandibular prominence; mx: maxillary prominence; ns: non-significant; pal: palate; sn: snout; tb: tooth bud; ton: tongue; vs: vessel. Scale bar in (A-C): 100 μm; scale bar in (A) applies to (M-Q); scale bars in others: 200 μm.

Next, we performed detailed phenotypic study on *Wnt1-cre;mTOR*^*loxp/loxp*^ cKO mice. The majority of *Wnt1-cre;mTOR*^*loxp/loxp*^ cKO mice died at E12.5, with very few survived to E14.5 (found at a ratio of 3%, expected at 25%). By E11.5, *Wnt1-cre;mTOR*^*loxp/loxp*^ cKO mice could be visually recognized by changes in craniofacial morphology. At this stage, growth of the five facial primordia in mutants was markedly delayed compared to controls, as evidenced by hypoplasia of mutant facial primordia (Fig 2A and 2B). The FN prominences failed to expand to the midline (Fig 2A and 2B). Thereafter, development of facial primordia was arrested and midline facial cleft persisted in mutants with full penetrance (Fig 2A, 2B and 2C). Histologic examination revealed defective organogenesis in multiple organ systems, including the palate, tongue, tooth, and skeleton (Fig 2D–2G). Despite of these defects, a considerable number of NCCs survived in mutant facial primordia, as indicated by distribution of GFP-labelled NCCs in the *Wnt1-cre*; *mTOR* ^*loxp/loxp*^; *Rosa* ^*nT-nG*^ mice (Fig 2B).

Bmp and Wnt signaling was obviously impaired in mutants, as indicated by reduced levels of p-Smad1/5/8 and β-catenin in NCC condensates (Fig 2H–2L). Staining of NCC marker *Alx3*, *Msx1*, and *Pax3* showed reduced expression in mutant facial primordia, suggesting that NCCs changed in gene expression (Fig 2M–2P and 2R). Expression of epithelial marker *Fgf8* remained comparable to the control, suggesting that epithelium development was virtually normal at this stage (Fig 2Q and 2R).

### Defective organogenesis in multiple systems in *mTOR* cKO mutant mice

**Fig 3:**
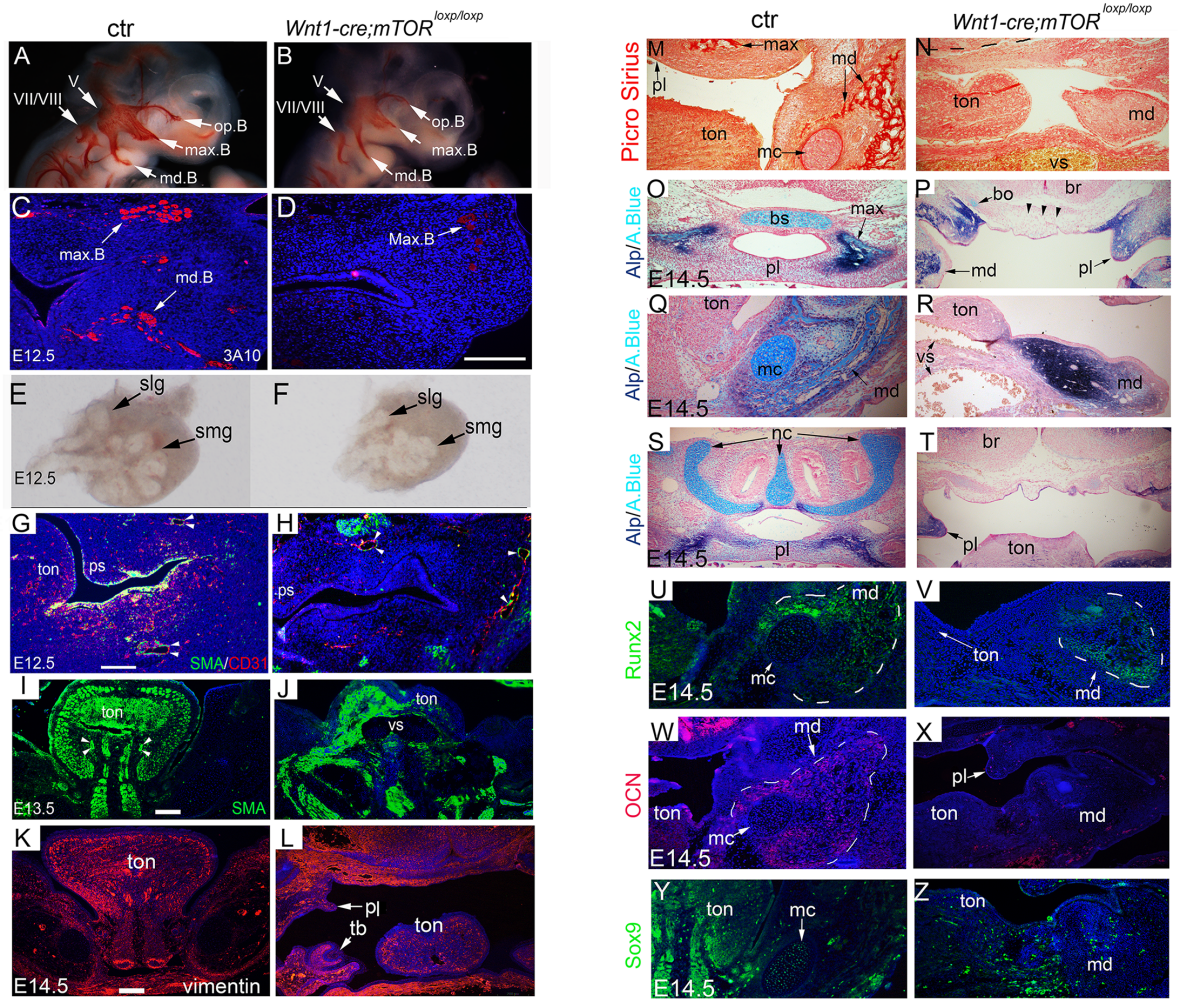
Defective organogenesis in multiple organ systems. (A, B) Whole mount staining for neurofilament marker 3A10 at E10.5. (C, D) Immunofluorescence for 3A10 at E12.5. (E, F) Development of submandibular glands at E12.5, examined under a stereomicroscope. (G, H) Immunofluorescence for CD31 and α-SMA at E12.5. (I, J) Immunofluorescence for α-SMA at E13.5. Note, severe dilation of the oro-craniofacial vessels in the mutant. (K, L) Immunofluorescence for vimentin at E14.5. (M, N) Picrosirius red staining at E14.5 reveals absence of maxilla and mandible in the mutant. (O-T) Alcian blue and ALP double staining shows absence of cartilages (arrow heads) and bones in mutants. (U-Z) Immunofluorescence for Runx2, OCN and Sox9 in the mandibular arch. A.Blue: alcian blue; bo: basioccipital cartilage; br: brain, bs: basosphenoid cartilage; ctr: control; mc: the Meckel’s cartilage; md: mandibular arch; md.B: mandible branch; ml: molar; max: maxilla; max.B: maxillary branch; oph.B: ophthalmic branch; pl (ps in G and H): palate; slg: sublingual gland; smg: submandibular gland; ton: tongue; vs: vessel. Scale bar in (A, B): 200 μm; scale bar in others: 100 μm; Scale bar in (K) applies to (M-Z).

NCCs also contribute to neural tissues [1–4]. Whole mount staining with a neurolfilament marker 3A10 revealed reduced dimensions of mutant trigeminal ganglion and its branches at E10.5 (Fig 3A and 3B). By E12.5, the mandibular branch of mutant trigeminal nerve failed to reach the anterior segment of the mandibular arch, while the maxillary branch showed fewer neural fibers in mutants than in controls (Fig 3C and 3D).

The salivary glands and teeth are formed via continuous epithelial-mesenchymal interactions, and the mesenchymal tissue is principally of NCC origin [1–4]. Here we found that development of submandibular and sublingual glands in *mTOR* mutants was delayed, as evidenced by smaller sizes and fewer numbers of epithelial branching events than controls (Fig 3E and 3F, S2 Fig). Molar development in mutants failed to proceed beyond the bud stage (S2 Fig).

NCCs express *Vegf* and regulate angiogenesis and vasculogenesis during craniofacial development [26]. Examination of craniofacial vasculature with endothelial marker CD31 revealed that vascular structures were abundant in controls but few in mutants at E12.5, suggesting that disruption of *mTOR* in NCCs causes microvascular rarefaction (Fig 3G and 3H). α-SMA staining showed that vascular SMC layers were often malformed and discontinuous in mutants, thus vessel dilation and bleeding were common (Fig 3I and 3J). These results indicate that mutant SMCs were functionally insufficient.

NCCs also regulate mesoderm-derived tissues via diverse signal pathways [27–30]. The tongue, which is largely composed of mesoderm-derived muscles, was malformed in mutants. Mutant tongue bud was hypoplastic and displayed a groove in the midline (Fig 2D–2G). α-SMA and vimentin staining revealed disorganized tongue muscles in mutants (Fig 3I–3M).

### Defective osteogenesis and chondrogenesis in *mTOR* mutants

Craniofacial bones and cartilages, including the upper and lower jaws, frontal bone, anterior cranial base (ethmoid and sphenoid bones), nasal cartilages, and the Meckel’s cartilage were all derived of NCCs [1–4]. Histology and picrosirius red staining failed to reveal any bone tissue in mutant face up to E14.5 (Figs 2F, 2G, 3M and 3N). Staining for osteogenic markers revealed that cells in mutant face were positive for early osteogenic marker alkaline phosphatase (ALP) and Runx2, but negative for terminal osteogenic marker osteocalcin (OCN) (Fig 3O–3X). These results together indicate that mutant NCCs that had committed to the osteogenic fate failed in terminal differentiation. Alcian blue staining showed that the NCC-derived cartilages, including the anterior basicranium, the Meckel’s cartilage and nasal cartilage, were either missing or extremely rudimentary (Fig 3O–3T). Furthermore, the master chondrogenesis marker Sox9 was not detected or barely detectable in mutant facial primordia, suggesting a failure of chondrogenic differentiation (Fig 3Y and 3Z).

### NCC migration and early PA morphogenesis were virtually normal in *mTOR* cKO mutants

**Fig 4:**
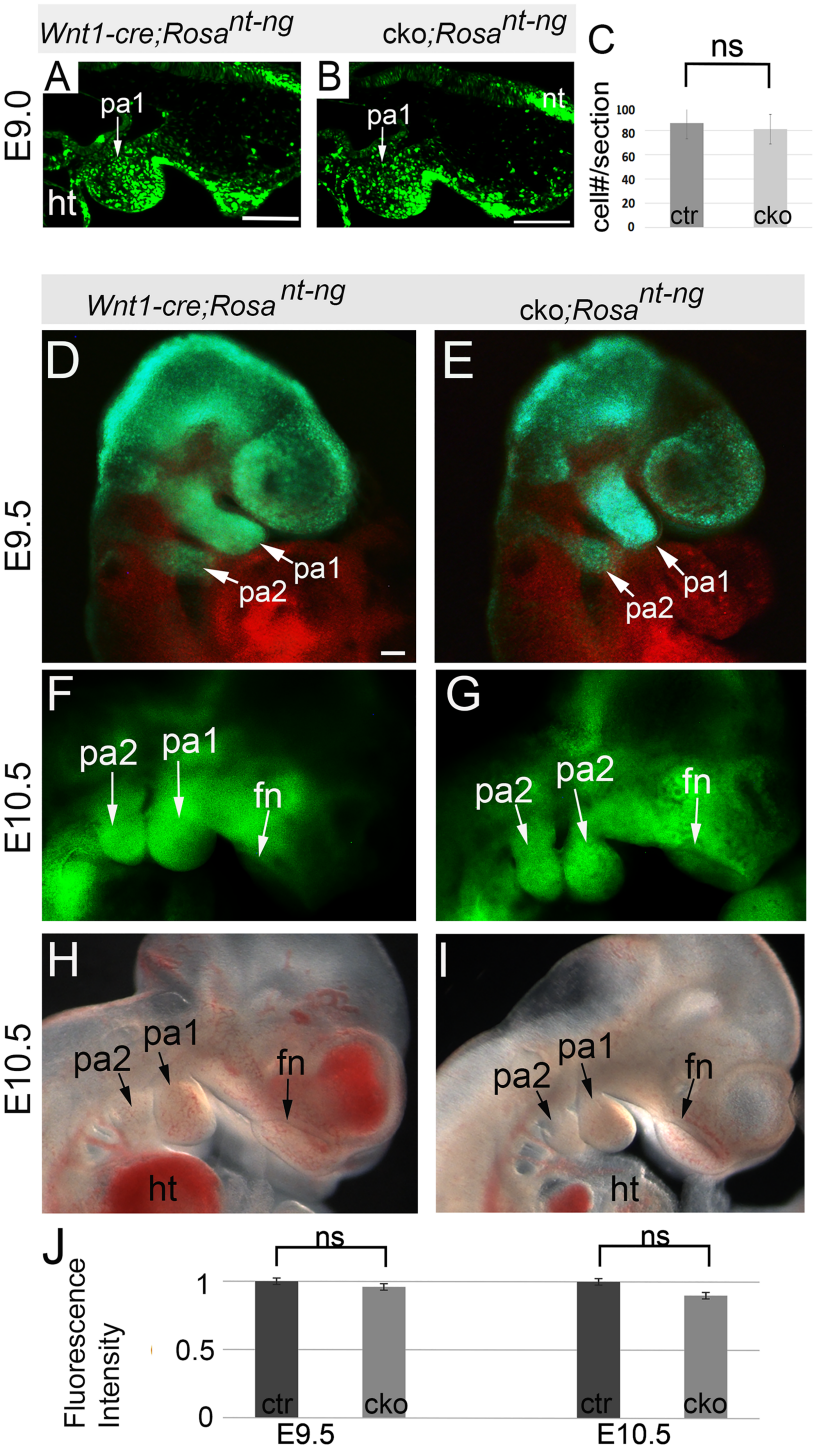
Initial NCC migration and PA morphogenesis. (A, B) Sagittal sections of E9.0 embryos (15-somite stage). (C) Quantification of the number of NCCs at E9.0 stage. (D, E) E9.5 embryos observed by a fluorescent microscope. Green fluorescence (GFP) labels NCCs; red fluorescence (tdTomato) labels non-NCCs. (F, G) E10.5 embryos observed by a fluorescent microscope. (H, I) Dark field observation of E10.5 embryos. (J) Quantification of GFP fluorescence intensity of E9.5 and E10.5 mice. fn: frontonasal prominence; ht: heart: ns: non-significant; pa: pharyngeal arch. Scale bars: 100 μm.

Next, we examined if NCC migration was affected in mutants using *Wnt1-cre*; *mTOR* ^*loxp/loxp*^; *Rosa* ^*nT-nG*^ mice, in which NCCs and their derivatives were GFP-labelled. At E9.0, when NCC migration had started, we observed comparable amounts of GFP-labeled NCCs on their migration paths and in the first pair of PAs in mutants and stage-matched littermate controls (Fig 4A–4C). Mice at E9.5 and E10.5 also displayed comparable NCC distribution in the PAs and facial primordia of the two genotypes (Fig 4D–4J). Altogether, these results indicate that initial migration and early development of cranial NCCs and PAs were virtually unaffected in mutants.

### Increased apoptosis and reduced cell proliferation in mutant facial primordia

**Fig 5:**
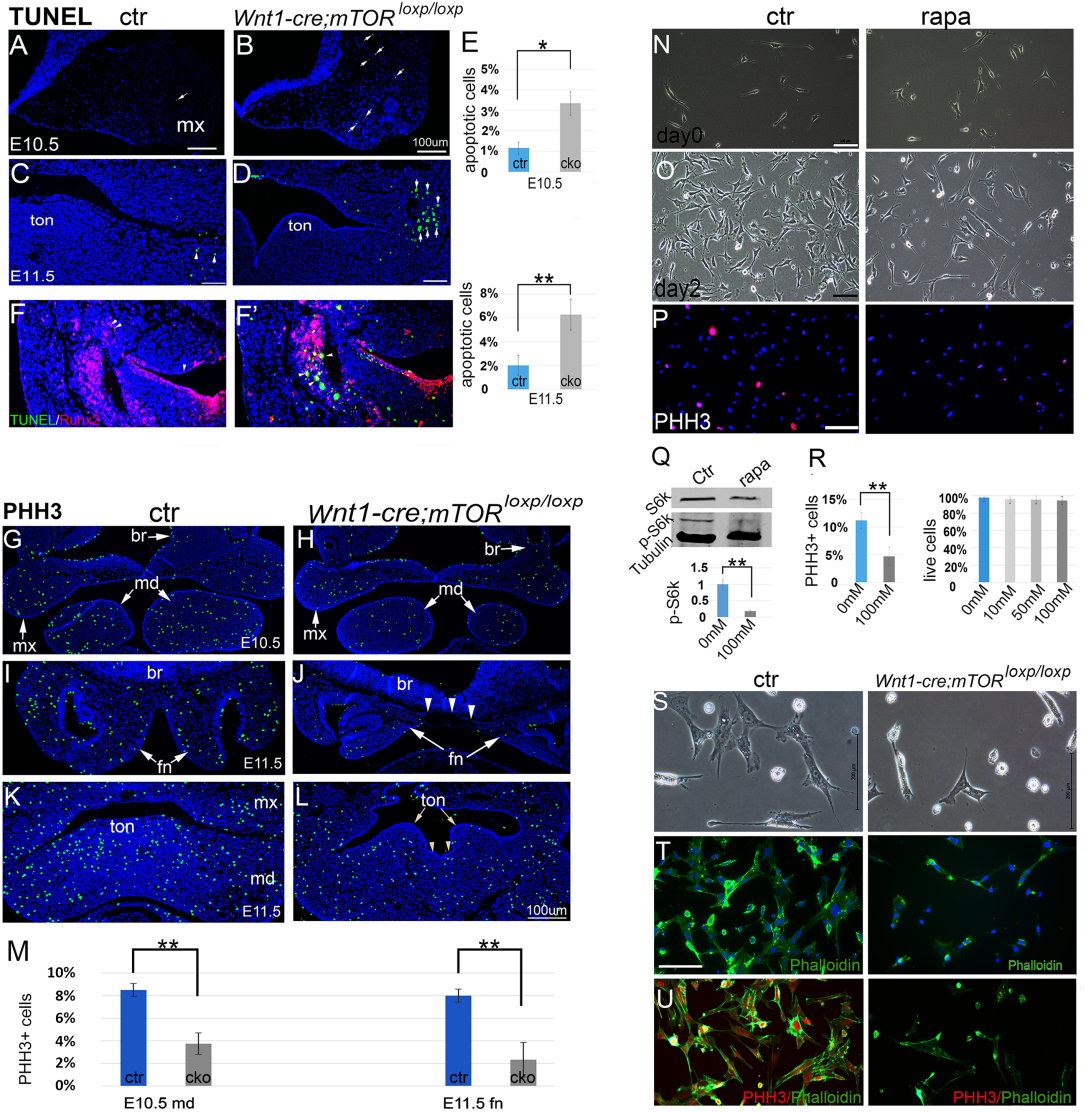
Apoptosis and proliferation assays. (A-D) Apoptosis at E10.5 and E11.5. (E) Quantification of apoptotic cells. (F, F’) Dual staining of TUNEL and Runx2 shows that apoptotic cells are predominantly NCC descendants. (G, H) Immunofluorescence for PHH3 in E10.5 facial primordia. (I, J) Immunofluorescence for PHH3 in the FN at E11.5. (K, L) Immunofluorescence for PHH3 in the mandibular arch at E11.5. Arrowheads indicate groove in the tongue. (M) Quantification of PHH3+ cells. (N, O) O9 NCCs cultured with and without rapamycin (100nM). (P) PHH3 staining of O9 cells. (Q)Western blot for mTORC1 downstream target p-S6K1/S6K1 upon rapamycin treatment. (R) Percentage of PHH3+ cells and cell live/death assay. (S) Phase contrast images of cultured PA cells. (T) Phalloidin and Dapi staining of PA cells. (U) PHH3 and Phalloidin double staining of PA cells. br: brain; ctr: control; fn: frontonasal prominence; md: mandibular prominence; mx: maxillary prominence; rapa: rapamycin; ton: tongue. Scale bars in (A-D): 100 μm.; scale bars in (L-U): 200 μm; scale bar in (L) applies to (G-K).

TUNEL staining revealed slightly increased apoptosis in mesenchyme of mutant facial primordia at E10.5 (Fig 5A and 5B). By E11.5, apoptosis was markedly increased in mutants (Fig 5C, 5D and 5E). By co-localization of apoptotic cells with Runx2+ cells, we demonstrated that apoptotic cells were principally NCC-derived cells that had been committed to the osteogenic fate (Fig 5F and 5F’).

Reduced cell proliferation was already obvious at E10.5 in mutant facial primordia, demonstrated by staining of a mitosis marker phospho-histone H3 (PHH3) (Fig 5G and 5H). At E11.5, very few mitotic cells were detected in mutant FNs and other facial primordia, relative to vigorous cell mitosis in controls (Fig 5I–5M). Moreover, we observed reduced cell proliferation in mutant tongue bud, which is largely composed of mesoderm-derived muscular precursors, suggesting that multiple cell lineages were affected (Fig 5K and 5L).

We also demonstrated the role of mTOR for cell proliferation in the NCC-derived O9 cells by using rapamycin, a well-characterized mTORC1 inhibitor, to suppress mTOR activity in NCCs in a culture system (Fig 5N–5R). Rapamycin treatment significantly reduced p-S6K levels in 24 hours, confirming its role of suppressing mTORC1 activity (Fig 5Q). O9 cells reached 80% confluence after two days in culture, whereas rapamycin-treated cells expanded slowly and reached only 50% confluence (Fig 5N–5P). Cell live/death analysis did not reveal a significant difference between the two groups (Fig 5R). PHH3 staining showed reduced percentage of mitotic cells in rapamycin treated cells, corroborating that cell proliferation was reduced upon rapamycin treatment (Fig 5R).

We further characterized primary NCCs isolated from the first pair of PAs of E9.5 mice. Consistent with in vivo observation, mTOR-deficient NCCs expanded slowly in culture and displayed altered morphology compared to controls (Fig 5S). Phalloidin and PHH3 staining revealed reduced cytoskeleton and decreased number of PHH3+ cells in mTOR-deficient cells (Fig 5T and 5U). Altogether, these results support that the mTOR pathway is critical for NCC proliferation and survival.

### Disruption of *mTOR* causes hyperactivity of P53 in the facial primordia and lowering P53 activity attenuates craniofacial phenotype in NCC-*mTOR* cKO mice

**Fig 6:**
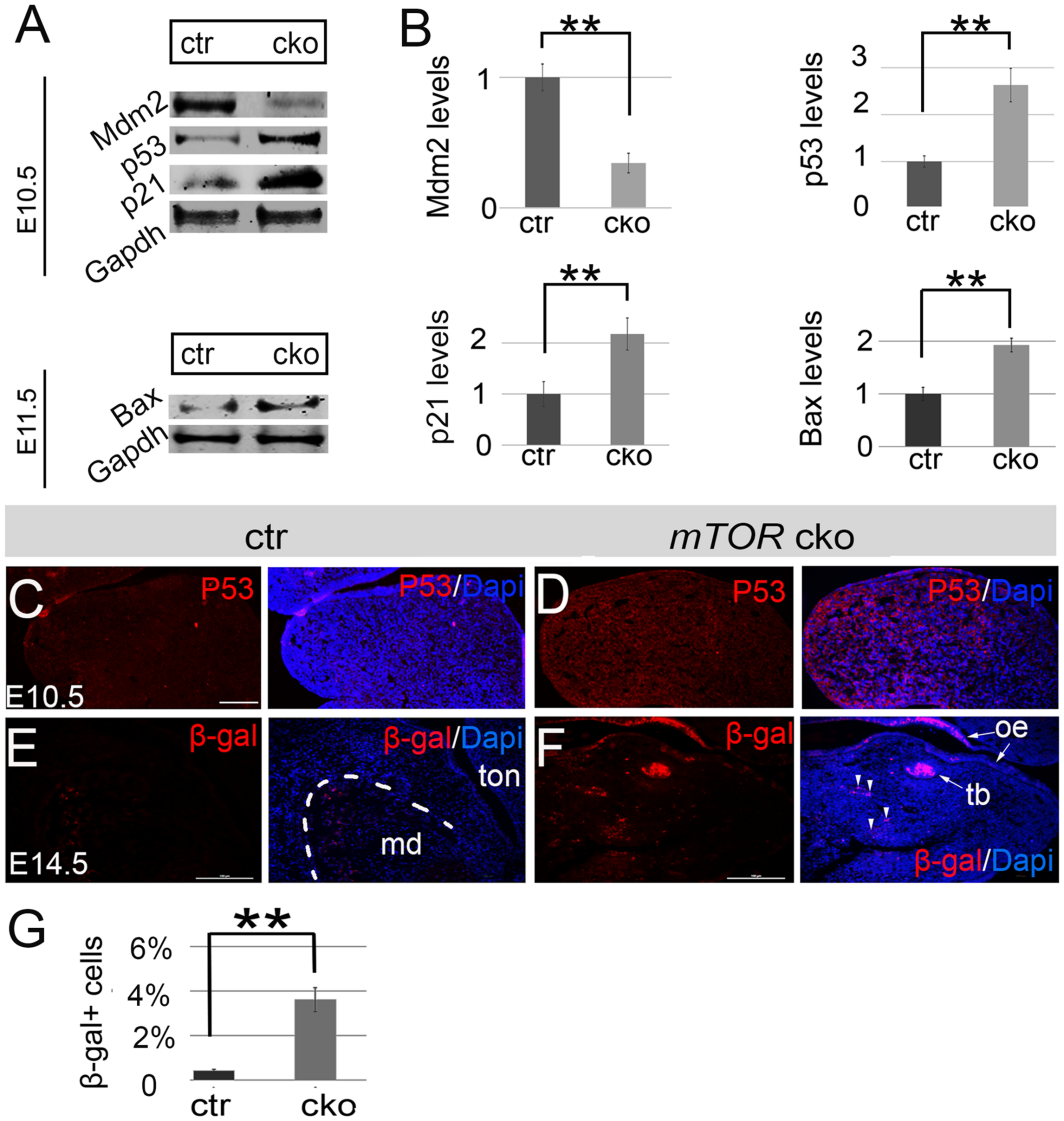
Hyperactivity of P53 signaling in the NCC-*mTOR* KO mice. (A) Western blots for Mdm2, P53, P21 and Bax of E10.5 and E11.5 facial primordia. (B) Quantification of western blots of Mdm2, P53, P21 and Bax. (C, D) Immunofluorescence for P53 in the mandibular prominence of E10.5 mice. (E, F) Immunofluorescence for β-galactosidase in the mandibular arch of E14.5 mice. Note, a number of positive cells are present in mutant mandibular arch. (G) Quantification of β-galactosidase positive cells. **P<0.01. β-gal: β-galactosidase; ctr: control; md: mandible; oe: oral epithelium; tb: tooth bud; ton: tongue. Scale bar: 100μm.

P53 is important for NCC development and P53 activity is elevated in a variety of developmental disorders [31–37]. Here we found that levels of P53 and its downstream factor P21 were markedly elevated in the facial primordia of NCC-*mTOR* cKO mice (Fig 6A–6D). Furthermore, levels of Mdm2, a negatively regulator of P53 through physical binding [38, 39], were significantly decreased (Fig 6A and 6B). Hyperactivity of P53 and P21 might account for the observed apoptosis and cell cycle arrest. Interestingly, we observed cytoplasmic accumulation of P53 in mutants, suggesting potential transcription-independent functions of P53 (Fig 6C and 6D). We therefore examined additional markers for the P53-dependent pathways including Bax and β-galactosidase, whose activities are associated with mitochondrial apoptosis (the intrinsic apoptosis pathway) and cell senescence respectively. Results showed that Bax levels were significantly elevated in mutants, implying that P53 induces cell death, at least in part, via the intrinsic apoptotic pathway (Fig 6A and 6B). β-galactosidase antibody staining failed to reveal any positive cell in mutant facial primordia up to the stage of E13.5, when craniofacial development had been arrested. By E14.5, however, we detected a number of β-galactosidase positive cells in mutant facial primordia, in contrast to virtually no positive cells in controls (Fig 6E, 6F and 6G). These results suggest that cell senescence was a late and uncommon event of mTOR deficiency.

**Fig 7:**
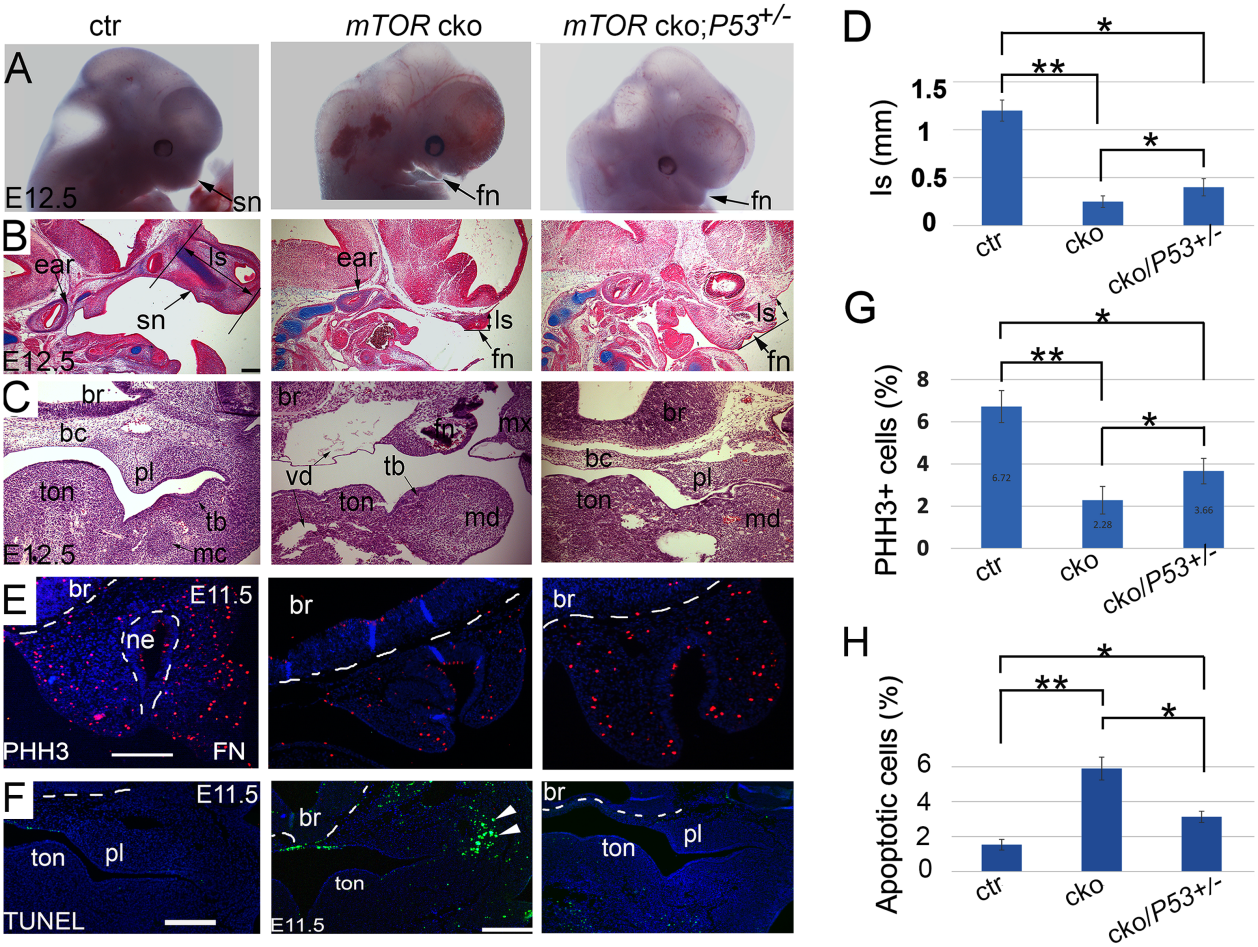
Lowering P53 activity by *P53* copy reduction attenuates the craniofacial phenotype in the NCC-*mTOR* cKO mice. (A) Gross examination of NCC-*mTOR* cKO mice by *P53* copy deduction. (B) Alcian blue staining in parasagittal sections of E12.5 heads. (C) Histology in frontal sections of E12.5 heads. (D) Quantification of the length of snout/frontonasal prominence, which is represented by the length from the brain-nose turning point to the most anterior plane of the snout. (E) PHH3 staining in frontal sections of E11.5 FN. (F) Apoptosis in frontal sections of E11.5 heads (arrow heads). (G, H) Quantification of cell proliferation and apoptosis. * P<0.05; **P<0.01. bc: basicranium; br; brain; ctr: control; fn: frontonasal prominence; ls: length of the snout; mc: Meckel’ cartilage; md: mandibular arch; mx: maxillary arch; ne: nasal epithelium; pl: palatal shelf; sn: snout; tb: tooth bud; ton: tongue; vd: vessel dilation. Scale bar: 200μm.

We next sought to determine if lowering P53 activity would be able to rescue the defects in NCC-*mTOR* cKO mice. We performed one copy reduction of *P53* in NCC-*mTOR* cKO mice by establishing the *Wnt1-Cre;mTOR*^*loxp/loxp*^; *P53*^+/-^ mice. Results showed that *P53* haplodeficiency caused a mild craniofacial phenotype in *mTOR* cKO mutants at E12.5, as evidenced by more pronounced snouts than *P53*^*+/+*^ mutants (Fig 7A). Sectional examination confirmed improved development of the snouts (Fig 7B, 7C and 7D). Development of the anterior basicranium was also ameliorated by a copy-reduction of *P53* (Fig 7C). However, midline growth deficiency and defective organogenesis persisted, i.e. missing of cartilages, grooved tongue and vascular dilation (Fig 7C). In addition, cell proliferation in the facial primordia, although increased compared to *P53*^*+/+*^ mutants, were not elevated to a normal level, implying that haplodeficiency of *P53* was not sufficient to fully rescue the proliferation defect in mutants (Fig 7E and 7G). Remarkably, the proportion of apoptotic cells in mTOR cKO mutants was significantly reduced by P53 haplodeficiency (Fig 7F and 7G).

### Dissecting functional roles of mTORC1 and mTORC2 for NCCs

**Fig 8:**
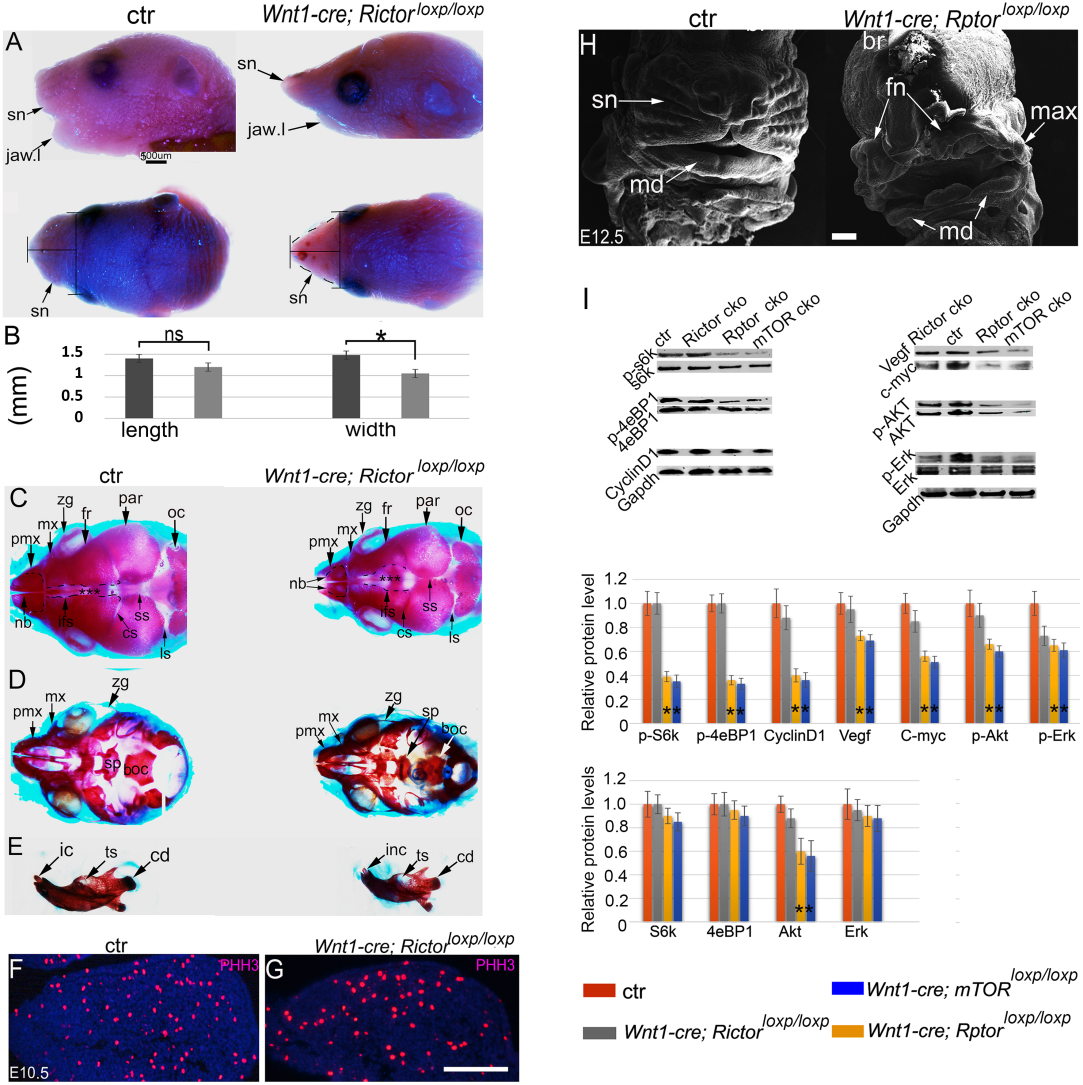
Craniofacial development of the NCC-*Rictor* and NCC-*Rptor* cKO mice. (A) Lateral and top view of mouse heads at the newborn stage. (B) Quantification of snout length (the most anterior point to the most anterior eye line) and width (the line at the mid-point of the length line), *p<0.05. (C, D, E) Skeleton preparation by alizarin red and alcian blue staining at the newborn stage, demonstrating hypoplasia of the craniofacial bones and an enlarged interfrontal suture in the mutant. (F, G) Immunofluorescence for PHH3 in the first PA. (H) SEM of the NCC-*Rptor* cKO mouse heads. (I) Western blots for p-S6K1,S6K1, p-4E-BP1, 4E-BP1, Cyclin D1, Vegf, c-Myc, p-Akt, Akt, p-Erk, Erk, and Gapdh of E11.5 facial primordia, * p<0.05. boc: basioccipital bone; cd: condyle of the mandible; cs: coronal suture; ctr: control; fr: frontal bone; ic: incisor; inf: interfrontal suture; is: lambdoid suture; mx (max in H): maxilla; pmx: premaxilla; nb: nasal bones; ns: non-significant; oc: occipital bone; par: parietal bone; sn: snout; jaw;l: lower jaw; sp: sphenoid bone; ss:sagittal suture; ts: tooth socket; zy: zygomatic arch. Scale bar in (A): 500μm; scale bars in (G.H): 100 μm.

To know relative contributions of mTORC1 and mTORC2 in NCCs, we continued to perform NCC knockout of *Rptor* (NCC-*Rptor* cKO) and *Rictor* (NCC-*Rictor* cKO), the core components in mTORC1 and mTORC2 respectively. The majority of the NCC-*Rictor* cKO mice died shortly after birth with relatively mild craniofacial defects. Mutant mouse developed a thin face with a small snout and a short lower jaw (Fig 8A and 8B). Skeleton preparation by alizarin red and alcian blue staining showed that mutant facial bones were smaller than controls (Fig 8C–8E). Development of NCC-derived frontal bones was retarded and the interfrontal sutures were enlarged in mutants (Fig 8C). The coronal and sagittal sutures, by contrast, were narrowed compared to controls (Fig 8C). Mandibular hypoplasia was evident (Fig 8E). However, examination at embryonic stages revealed no obvious change in gross morphology, cell proliferation and apoptosis during craniofacial development (Fig 8F and 8G).

By sharp contrast, the majority of the NCC-*Rptor* cKO mice died at E12.5, exhibiting a full spectrum of defects in craniofacial morphogenesis and organogenesis phenocopying these seen in NCC-*mTOR* cKO mice (Fig 8H, and S3 Fig). These two mutant genotypes showed markedly reduced activation of the mTORC1 downstream factor S6K1 and 4e-BP1 from the facial primordia (Fig 8I). Protein levels of a set of cell cycle and signaling molecules, including Cyclin D1, c-Myc, p-Akt/Akt, p-Erk and Vegf were all reduced (Fig 8I). Altogether, these genetic models demonstrated that mTOR principally acts through mTORC1 for cranial NCC development during embryogenesis.

## Discussion

The functions and the underlying regulatory network of mTOR for NCC development remains to be elucidated. Multiple lines of evidence suggest a potential link of the mTOR and P53 pathways in diverse settings [34, 35, 40–42]. In a variety of contexts, P53 suppresses mTOR activity and inhibits cell proliferation [34]. In cranial NCC development, maintaining a proper level of P53 is of critical importance [31, 43]. Hyperactivity of P53 and lowered function of mTOR have been demonstrated in a number of human genetic diseases, such as the Roberts syndrome and Treacher Collins syndrome, which are featured by facial hypoplasia due to a cell proliferation defect [32, 33]. On the other hand, mTOR activation and P53 dysfunction are common events in the development of many types of cancers [44]. Elevated P53 levels in our mTOR cKO model imply that there may also exist a negative feedback loop between the mTOR and P53 pathways.

Although it has been firmly established that P53 inhibits the mTOR pathway through regulation of TSC1/TSC2, both of which are negative regulators of mTOR^5, 36, 40, 41^, the mechanism by which mTOR feedbacks to P53 remains to be revealed. Although reduced levels of P53 inhibitory regulators, such as Pax3, Mdm2 and p-Akt, may in part account for the P53 stabilization and accumulation in mutant mice [38, 39, 45], potential cellular stresses caused by *mTOR* deletion may be able to directly activate P53. Multiple lines of evidence point to a possible convergence of the mTOR and P53 pathways in the process of ribosome biogenesis, in which mTOR plays a critical role and whose stress causes P53 activation [6–9, 46].

Activation of P53 explains the upregulation of P21 and Bax, which further induces cell cycle arrest and activation of the intrinsic apoptosis pathway. Apoptosis, although increased in mutants, was only rampant at a stage when defective morphogenesis was already obvious. Thus, increased apoptosis is not likely a primary cause for abnormal development. Rather, it may be secondary to the cellular defects of nutrition acquiring, signal responding to the local developmental niche, and cellular stresses caused by mTOR deficiency. Despite of large numbers of cell death, a considerable amount of NCCs survived in the mutant facial primordia and entered into cell cycle arrest. Reduced apoptosis in P53 haplodeficincy mutants further corroborates that mTOR regulates survival of NCCs, at least in part, through interacting with the p53-dependent pathways.

However, lowering P53 activity, although promoted cell proliferation, failed to fully rescue the proliferation defect, and midline cleft remained in all mutants. β-galactosidase staining indicates that the majority of mutant NCCs were not yet in a senescence status. These results together point to the fact that additional mechanisms may also be at play accounting for the observed phenotype. Indeed, we found that levels of a number of important signaling and cell cycle molecules were reduced in mutants, including Vegf, Cyclin D1 and c-Myc. Individual NCC knockout of the genes that encode these proteins show mild craniofacial abnormalities compared to the NCC-*mTOR* cKO mice, suggesting that the phenotype in NCC-*mTOR* cKO mice is a combinational effect of multiple protein level changes [26, 47, 48].

In addition to these firmly established roles for cell proliferation and survival, mTOR is also critical for NCC differentiation. A major fate for cranial NCCs is to differentiate into skeletal cells including chondroblasts and osteoblasts, which form cartilages and bones respectively during craniofacial development. In NCC-*mTOR* cKO mice, NCC-derived cartilages, including the nasal cartilage, Meckel’s cartilage and anterior basicranium, were absent. We even failed to detect the chondrogenic marker Sox9 in the facial primordia of NCC-*mTOR* cKO mice, suggesting that mTOR is essential for chondrogenic differentiation of NCCs. This is in stark contrast with a previous study, showing the presence of cartilage precursors for endochondral bones of mesoderm origins [10]. Thus, mTOR is differently required for chondrogenic differentiation by NCCs and mesoderm-derived mesenchymal cells.

In contrast to chondrogenesis, NCC-*mTOR* cKO mice did show osteogenic differentiation in the facial primordia, as indicated by positive staining of Runx2 and ALP although at reduced levels. This implies that the roles of mTOR for cell differentiation depend on a specific context. However, bone matrix, which was abundant in controls, was barely detectable in mutants, suggesting that mutant osteoblasts failed in collagen synthesis. This is consistent with the observation in mesoderm-derived bones in a previous study [10]. Critical functions of mTOR for osteogenesis are further corroborated by an mTOR hyperactivity model, in which excessive bone formation and bone sclerosis were observed in NCC-derived bones [23].

NCCs regulate angiogenesis and in situ vascularization in NCC-derivatives through expressing *Vegf* and by directly differentiating into SMCs [26]. In NCC-*mTOR* cKO mice, α-SMA+ cells appeared functionally insufficient and often failed to form a complete SMC layer for the vasculature. As a result, vessel dilation and bleeding due to rupture were common. However, vascular defects were only detectable from the stage of late E11.5, when retarded facial development was already apparent. These results suggest that altered craniofacial morphogenesis is a primary defect occurring prior to vascular abnormality.

By analysis of *Rptor* and *Rictor* NCC cKO models, we demonstrate NCC-*Rptor* disruption caused a spectrum of defects mirroring that of the NCC-*mTOR* deletion, suggesting that mTOR mainly acts through mTORC1 for embryonic craniofacial development. By contrast, functions of mTORC2 are not critical for cell proliferation, survival and differentiation, and are dispensable for early craniofacial morphogenesis and growth. However, the mild craniofacial phenotype of mTORC2 disruption mice at the postnatal stage suggests that mTORC2 plays a role in regulating the size of craniofacial skeletons, and may have other unknown functions in maintaining tissue homeostasis. Hypoplasia of craniofacial skeletons seen in mTORC2 disruption mice is a hallmark in a variety of human genetic disorders [32, 33]. The roles of mTORC2 for postnatal growth and homeostasis of craniofacial organs are worthy of further investigation.

In summary, in this study we unveil critical roles of mTOR for NCC development by phenotypic analysis of multiple genetic models of the mTOR pathway. We show that mTOR is required for multiple developmental processes of post-migratory NCCs, including proliferation, survival and differentiation in a variety of organ systems and by regulating a number of signaling pathways. Significantly, we reveal that hyperactivity of P53 serves as an important mechanism among others underpinning abnormal craniofacial development. We also demonstrate that mTOR acts principally through the mTORC1 pathway for embryonic craniofacial development and growth.

## Materials and methods

### Ethics statement

Mouse experiments were approved by Columbia University Institutional Animal Care and Use Committee (protocol number AC-AAAS0552.

### Generation of tissue specific knockout models for mTOR, mTORC1 and mTORC2

*Wnt1-cre* (No.022501) and *mTOR* ^*loxp/loxp*^ (No.011009), *Rictor* ^*loxp/loxp*^ (No.020649), *Rptor* ^*loxp/loxp*^ (No. 013188), *P53*^*+/-*^ (002101) and *Rosa* ^*nT-nG*^ (No.023035) mice were purchased from The Jackson Laboratory [18, 24, 49–51]. *Rosa26-LacZ* mouse was described elsewhere [52]. Mouse genotyping was performed by general PCR procedures, using GoTaq Green Master Mix (Promega, Catalog number M7122). We used littermate wild type mice as controls unless otherwise described.

### SEM, histology, immunofluorescence, and in situ hybridization

Embryos for SEM were fixed in 2.5% glutaldehyde and dehydrated in a series of graded serial ethanol solutions. Frontal sections of paraffin-embedded tissues were used in this study unless otherwise described. Frozen sections were used for NCC lineage tracing and Alp staining. ALP-stained sections were co-stained with alcian blue. Specifically, sections were incubated in ALP buffer for 15 minutes and then incubated in a NBT/BCIP solution (Thermo Fisher Scientific, Catalog number 4072) until the color reaction was desirable. The sections were next stained with an alcian blue. Whole mount LacZ staining was performed according to previously described procedures, lacZ-stained embryos were sectioned and count-stained with Nuclear Fast Red solution (Vector, catalog number H-3403) [28]. Picrosirius red staining was performed following the manufacturer’s protocol (Abcam, catalog number ab150681).

Heat-induced antigen retrieval was applied for immunofluorescence. Deparaffinized sections were heated in 1x antigen unmasking solution (Vector, catalog number H-3301) at 100 °C for 45 minutes. Antibodies for phospho-mTOR (phospho-S2448; Abcam, ab109268), AP-2α (Abcam, ab189995), beta-catenin (Abcam, ab6302), p-Smads1/5/8 (Millipore, AB3848-I), Runx2 (Abcam, ab76956), OCN (Abcam, ab93876), Sox9 (Abcam, ab26414), α-SMA (Abcam, ab124964), CD31 (Abcam, ab7388), 3A10 (DSHB, supernatant), Vimentin (Abcam, ab92547), pan-keratin (Abcam, ab8068), P53 (Santa cruz, sc-71819), and β-galactosidase (Invitrogen, 14-6773-81) were used. Alexa Fluror 488 and Alexa Fluror 555 secondary antibodies were used for signal detection (Thermo Fisher Scientific). Antibody dilation followed the manufacturer’ s recommendations. Primary antibody was incubated overnight at 4 °C, secondary antibody was incubated at room temperature for 2 hours. Fluorescence signaling quantification was performed by calculating average fluorescence intensity of the signal channels using the Image J software (National Institutes of Health) in accordance with the recommended guidelines.

Whole mount in situ hybridization was performed as previously described [28]. Digoxigenin-labeled riboprobes of *Alx3*, *Msx1*, *Pax3* and *Fgf8* were generated by in vitro transcription using a kit from Roche (Catalog number 10999644 001) [28]. A NBT/BCIP solution was used for color reaction. Each probe staining was performed at least on three embryos of each genotype. Staining of the images were quantified with image J. Stained areas were selected by threshold adjustment of the signal channels.

### Apoptosis and proliferation assay

Paraffin sections were used for TUNEL staining and proliferation assays. TUNEL staining for apoptosis was performed using the DeadEnd TUNEL System (Promega, G3250), following the manufacturer’s protocol. The fraction of apoptotic cells over total number of cells was used for apoptosis index. PHH3 staining was used for proliferation assay (Millipore, 06–570). The fraction of PHH3 positive cells over total number of cells was used for proliferation index. Representative and comparable sections from three independent samples of each genotype were used for assessment.

### Cranial NCC culture

Primary NCCs were isolated from the first PA of E9.5 mice. Briefly, the first pair of PAs were dissected off and digested with Dispase to remove the epithelia. Mesenchyme was further digested with a collagenase solution, and cultured in a stem cell medium containing 15% serum and 100ng/ml Fgf2 (Millipore, GF003). O9 cells were purchased from the Millipore and maintained following manufacturer’s recommendations [53]. Approximately 50,000 cells were seeded per well in a 6-well plate. Live/death assay was performed using a detection kit following manufacturer’s protocol (Thermo Fisher, L3224). A serial of concentrations of rapamycin (Cell signaling, catalog number 9904) (10nM, 50nM, and 100nM) were applied. All experiments were performed in triplicates.

### Western blot assay

Cell lysates for western blots were prepared from cultured NCCs. Specifically, NCCs, seeded in 6-well plates, were collected and lysed with RIPA buffer for half hour at room temperature and then centrifuged at 11500 rpm for 20 minutes. Supernatants were used for western blots. Lysates of mouse samples were prepared from E10.5 and E11.5 facial primordia. The facial primordia of two embryos were dissected off, homogenized, lysed in 100μm RIPA buffer for 1 hour, and centrifuged at 11500 rpm for 20 minutes. Supernatants were collected for western blots, and 25μl lysate per sample was loaded per lane. Antibodies for p-S6K (phospho-T389; Abcam, ab2571), S6K (Abcam, ab32529), p-4eBP1 (phospho-Thr 45; Santa cruz, sc-271947), 4eBP1 (Santa cruz, sc-9977), Vefg (Santa cruz, sc7269), p-Akt1 (phospho-Thr 308; Santa cruz, sc-135650), Akt1(Santa cruz, sc-135829), p-Erk1/2 (phospho-Thr 202 and phospho-Tyr 204; Santa cruz, sc-81492), Erk1/2 (Santa cruz, sc-135900), c-Myc (Abcam, ab32072), P21 (Santa cruz, sc-6246), P53 (Santa cruz, sc-71819), Mdm2 (Santa cruz, sc-965), Bax (Santa cruz, sc-23959), β-tubulin (Santa cruz, sc-101527) and Gapdh (Santa cruz, sc-47724) were used. IRDye secondary antibodies were used for signal detection (Abcam). Antibody dilation followed manufacturer’s recommendations. All experiments were repeated 3 times each. Western blot bands were quantified with Image J.

### Statistical analysis

Quantification of tissue sections were performed by analyzing three independent biological samples, two representative and comparable sections for each. Quantification of western blot was performed by analyzing three technical replicates. Quantification was performed by two-tailed t-test and one-way ANOVA with post-hoc tests using Microsoft Excel software. Data was presented as means and standard deviation. P<0.05 was regarded as statistically significant.

## Supporting information

S1 Fig mTOR expression at successive stages. (A, B) Immunofluorescence for p-mTOR of the first PA at E9.5. (C, D) Immunofluorescence for p-mTOR at E11.5. (E, F) Immunofluorescence for p-mTOR at E14.5. br: brain; mc: Meckel’ s cartilage; md: mandible; msc: mesenchymal condensate; nf: neurofilament; pa: pharyngeal arch; tb: tooth bud; ton: tongue. Scale bar: 100 μm.
(TIF)

S2 Fig Defective development of the submandibular gland and mandibular first molar. (A, B) Gross examination of the submandibular gland with a fluorescent microscope at E13.5. (C, D) Mandibular first molar stained with pan-keratin. Development of mutant molar is arrested at the bud stage. pan-k: pan-keratin; m1: the first molar; smg: submandibular gland. Scale bar: 100μm.
(TIF)

S3 Fig Defective craniofacial development in NCC-*Rptor* cKO mice. (A, B) Lateral view of E11.5 embryos. (C-F) Lateral and frontal view of E13.5 embryos. Arrowheads in (F) indicate midline cleft in the mutant. (G) Quantification of P53 levels of E11.5 mouse facial primordia, *P<0.05. (H, I) PHH3 staining at E10.5. (J, K) Apoptosis at E11.5. (L) Quantification of PHH3+ cells and apoptotic cells, **p<0.01. br: brain; fn: frontonasal prominence; md: mandibular prominence; mx: maxillary prominence: pal: palate; sn: snout; ton: tongue. Scale bar (A-F): 500 μm; (G-J): 100 μm.
(TIF)

## References

[1] Simoes-Costa M, Bronner ME. Establishing neural crest identity: a gene regulatory recipe. Development. 2015;142(2):242–57. 10.1242/dev.105445 . 25564621 PMC4302844

[2] Bronner ME, Simoes-Costa M. The Neural Crest Migrating into the Twenty-First Century. Curr Top Dev Biol. 2016;116:115–34. 10.1016/bs.ctdb.2015.12.003 . 26970616 PMC5100668

[3] Achilleos A, Trainor PA. Neural crest stem cells: discovery, properties and potential for therapy. Cell Res. 2012;22(2):288–304. 10.1038/cr.2012.11 . 22231630 PMC3271580

[4] Mayor R, Theveneau E. The neural crest. Development. 2013;140(11):2247–51. 10.1242/dev.091751 . 23674598

[5] Chai Y, Jiang X, Ito Y, Bringas P Jr., Han J, Rowitch DH, et al Fate of the mammalian cranial neural crest during tooth and mandibular morphogenesis. Development. 2000;127(8):1671–9. . 10725243 10.1242/dev.127.8.1671

[6] Laplante M, Sabatini DM. Regulation of mTORC1 and its impact on gene expression at a glance. J Cell Sci. 2013;126(Pt 8):1713–9. 10.1242/jcs.125773 . 23641065 PMC3678406

[7] Laplante M, Sabatini DM. mTOR signaling in growth control and disease. Cell. 2012;149(2):274–93. 10.1016/j.cell.2012.03.017 . 22500797 PMC3331679

[8] Laplante M, Sabatini DM. mTOR signaling at a glance. J Cell Sci. 2009;122(Pt 20):3589–94. 10.1242/jcs.051011 . 19812304 PMC2758797

[9] Hay N, Sonenberg N. Upstream and downstream of mTOR. Genes Dev. 2004;18(16):1926–45. 10.1101/gad.1212704 . 15314020

[10] Chen J, Long F. mTORC1 signaling controls mammalian skeletal growth through stimulation of protein synthesis. Development. 2014;141(14):2848–54. 10.1242/dev.108811 . 24948603 PMC4197614

[11] Alessi DR, Pearce LR, Garcia-Martinez JM. New insights into mTOR signaling: mTORC2 and beyond. Sci Signal. 2009;2(67):pe27 10.1126/scisignal.267pe27 . 19383978

[12] Guertin DA, Stevens DM, Thoreen CC, Burds AA, Kalaany NY, Moffat J, et al Ablation in mice of the mTORC components raptor, rictor, or mLST8 reveals that mTORC2 is required for signaling to Akt-FOXO and PKCalpha, but not S6K1. Dev Cell. 2006;11(6):859–71. 10.1016/j.devcel.2006.10.007 . 17141160

[13] Oh WJ, Jacinto E. mTOR complex 2 signaling and functions. Cell Cycle. 2011;10(14):2305–16. 10.4161/cc.10.14.16586 . 21670596 PMC3322468

[14] Gangloff YG, Mueller M, Dann SG, Svoboda P, Sticker M, Spetz JF, et al Disruption of the mouse mTOR gene leads to early postimplantation lethality and prohibits embryonic stem cell development. Mol Cell Biol. 2004;24(21):9508–16. 10.1128/MCB.24.21.9508-9516.2004 . 15485918 PMC522282

[15] Shiota C, Woo JT, Lindner J, Shelton KD, Magnuson MA. Multiallelic disruption of the rictor gene in mice reveals that mTOR complex 2 is essential for fetal growth and viability. Dev Cell. 2006;11(4):583–9. 10.1016/j.devcel.2006.08.013 . 16962829

[16] Chen J, Holguin N, Shi Y, Silva MJ, Long F. mTORC2 signaling promotes skeletal growth and bone formation in mice. J Bone Miner Res. 2015;30(2):369–78. 10.1002/jbmr.2348 . 25196701 PMC4322759

[17] Ka M, Condorelli G, Woodgett JR, Kim WY. mTOR regulates brain morphogenesis by mediating GSK3 signaling. Development. 2014;141(21):4076–86. 10.1242/dev.108282 . 25273085 PMC4302893

[18] Risson V, Mazelin L, Roceri M, Sanchez H, Moncollin V, Corneloup C, et al Muscle inactivation of mTOR causes metabolic and dystrophin defects leading to severe myopathy. J Cell Biol. 2009;187(6):859–74. 10.1083/jcb.200903131 . 20008564 PMC2806319

[19] Land SC, Scott CL, Walker D. mTOR signalling, embryogenesis and the control of lung development. Semin Cell Dev Biol. 2014;36:68–78. 10.1016/j.semcdb.2014.09.023 . 25289569

[20] Ge Y, Chen J. Mammalian target of rapamycin (mTOR) signaling network in skeletal myogenesis. J Biol Chem. 2012;287(52):43928–35. 10.1074/jbc.R112.406942 . 23115234 PMC3527976

[21] Yu JS, Cui W. Proliferation, survival and metabolism: the role of PI3K/AKT/mTOR signalling in pluripotency and cell fate determination. Development. 2016;143(17):3050–60. 10.1242/dev.137075 . 27578176

[22] Bockaert J, Marin P. mTOR in Brain Physiology and Pathologies. Physiol Rev. 2015;95(4):1157–87. 10.1152/physrev.00038.2014 . 26269525

[23] Fang F, Sun S, Wang L, Guan JL, Giovannini M, Zhu Y, et al Neural Crest-Specific TSC1 Deletion in Mice Leads to Sclerotic Craniofacial Bone Lesion. J Bone Miner Res. 2015;30(7):1195–205. 10.1002/jbmr.2447 . 25639352 PMC4478231

[24] Lewis AE, Vasudevan HN, O’Neill AK, Soriano P, Bush JO. The widely used Wnt1-Cre transgene causes developmental phenotypes by ectopic activation of Wnt signaling. Dev Biol. 2013;379(2):229–34. 10.1016/j.ydbio.2013.04.026 . 23648512 PMC3804302

[25] Zhao H, Bringas P Jr., Chai Y. An in vitro model for characterizing the post-migratory cranial neural crest cells of the first branchial arch. Dev Dyn. 2006;235(5):1433–40. 10.1002/dvdy.20588 . 16245337 PMC3337696

[26] Wiszniak S, Mackenzie FE, Anderson P, Kabbara S, Ruhrberg C, Schwarz Q. Neural crest cell-derived VEGF promotes embryonic jaw extension. Proc Natl Acad Sci U S A. 2015;112(19):6086–91. 10.1073/pnas.1419368112 . 25922531 PMC4434710

[27] Iwata J, Suzuki A, Pelikan RC, Ho TV, Chai Y. Noncanonical transforming growth factor beta (TGFbeta) signaling in cranial neural crest cells causes tongue muscle developmental defects. J Biol Chem. 2013;288(41):29760–70. 10.1074/jbc.M113.493551 . 23950180 PMC3795273

[28] Nie X, Deng CX, Wang Q, Jiao K. Disruption of Smad4 in neural crest cells leads to mid-gestation death with pharyngeal arch, craniofacial and cardiac defects. Dev Biol. 2008;316(2):417–30. 10.1016/j.ydbio.2008.02.006 . 18334251 PMC2362382

[29] Rios AC, Serralbo O, Salgado D, Marcelle C. Neural crest regulates myogenesis through the transient activation of NOTCH. Nature. 2011;473(7348):532–5. 10.1038/nature09970 . 21572437

[30] Rinon A, Lazar S, Marshall H, Buchmann-Moller S, Neufeld A, Elhanany-Tamir H, et al Cranial neural crest cells regulate head muscle patterning and differentiation during vertebrate embryogenesis. Development. 2007;134(17):3065–75. 10.1242/dev.002501 . 17652354

[31] Rinon A, Molchadsky A, Nathan E, Yovel G, Rotter V, Sarig R, et al p53 coordinates cranial neural crest cell growth and epithelial-mesenchymal transition/delamination processes. Development. 2011;138(9):1827–38. 10.1242/dev.053645 . 21447558

[32] Xu B, Lee KK, Zhang L, Gerton JL. Stimulation of mTORC1 with L-leucine rescues defects associated with Roberts syndrome. PLoS Genet. 2013;9(10):e1003857 Epub 2013/10/08. 10.1371/journal.pgen.1003857 . 24098154 PMC3789817

[33] Jones NC, Lynn ML, Gaudenz K, Sakai D, Aoto K, Rey JP, et al Prevention of the neurocristopathy Treacher Collins syndrome through inhibition of p53 function. Nature Medicine. 2008;14(2):125–33. 10.1038/nm1725 18246078 PMC3093709

[34] Feng Z, Zhang H, Levine AJ, Jin S. The coordinate regulation of the p53 and mTOR pathways in cells. Proc Natl Acad Sci U S A. 2005;102(23):8204–9. 10.1073/pnas.0502857102 . 15928081 PMC1142118

[35] Moumen A, Patane S, Porras A, Dono R, Maina F. Met acts on Mdm2 via mTOR to signal cell survival during development. Development. 2007;134(7):1443–51. 10.1242/dev.02820 . 17329361

[36] Ye P, Liu Y, Chen C, Tang F, Wu Q, Wang X, et al An mTORC1-Mdm2-Drosha axis for miRNA biogenesis in response to glucose- and amino acid-deprivation. Mol Cell. 2015;57(4):708–20. 10.1016/j.molcel.2014.12.034 . 25639470 PMC4511160

[37] Van Nostrand JL, Brady CA, Jung H, Fuentes DR, Kozak MM, Johnson TM, et al Inappropriate p53 activation during development induces features of CHARGE syndrome. Nature. 2014;514(7521):228–32. 10.1038/nature13585 . 25119037 PMC4192026

[38] Ogawara Y, Kishishita S, Obata T, Isazawa Y, Suzuki T, Tanaka K, et al Akt enhances Mdm2-mediated ubiquitination and degradation of p53. J Biol Chem. 2002;277(24):21843–50. Epub 2002/03/30. 10.1074/jbc.M109745200 . 11923280

[39] Shi D, Gu W. Dual Roles of MDM2 in the Regulation of p53: Ubiquitination Dependent and Ubiquitination Independent Mechanisms of MDM2 Repression of p53 Activity. Genes Cancer. 2012;3(3–4):240–8. Epub 2012/11/15. 10.1177/1947601912455199 . 23150757 PMC3494363

[40] Maki CG. Decision-making by p53 and mTOR. Aging (Albany NY). 2010;2(6):324–6. 10.18632/aging.100166 . 20603526 PMC2919249

[41] Mungamuri SK, Yang X, Thor AD, Somasundaram K. Survival signaling by Notch1: mammalian target of rapamycin (mTOR)-dependent inhibition of p53. Cancer Res. 2006;66(9):4715–24. 10.1158/0008-5472.CAN-05-3830 . 16651424

[42] Fantauzzo KA, Soriano P. PI3K-mediated PDGFRalpha signaling regulates survival and proliferation in skeletal development through p53-dependent intracellular pathways. Genes Dev. 2014;28(9):1005–17. Epub 2014/05/03. 10.1101/gad.238709.114 . 24788519 PMC4018488

[43] Jones NC, Lynn ML, Gaudenz K, Sakai D, Aoto K, Rey JP, et al Prevention of the neurocristopathy Treacher Collins syndrome through inhibition of p53 function. Nat Med. 2008;14(2):125–33. 10.1038/nm1725 . 18246078 PMC3093709

[44] Kong B, Cheng T, Qian C, Wu W, Steiger K, Cao J, et al Pancreas-specific activation of mTOR and loss of p53 induce tumors reminiscent of acinar cell carcinoma. Mol Cancer. 2015;14:212 10.1186/s12943-015-0483-1 . 26683340 PMC4683950

[45] Wang XD, Morgan SC, Loeken MR. Pax3 stimulates p53 ubiquitination and degradation independent of transcription. PLoS One. 2011;6(12):e29379 10.1371/journal.pone.0029379 . 22216266 PMC3247257

[46] Calo E, Gu B, Bowen ME, Aryan F, Zalc A, Liang J, et al Tissue-selective effects of nucleolar stress and rDNA damage in developmental disorders. Nature. 2018;554(7690):112–7. Epub 2018/01/25. 10.1038/nature25449 . 29364875 PMC5927778

[47] Wei K, Chen J, Akrami K, Galbraith GC, Lopez IA, Chen F. Neural crest cell deficiency of c-myc causes skull and hearing defects. Genesis. 2007;45(6):382–90. 10.1002/dvg.20304 . 17523175

[48] Fantl V, Stamp G, Andrews A, Rosewell I, Dickson C. Mice lacking cyclin D1 are small and show defects in eye and mammary gland development. Genes Dev. 1995;9(19):2364–72. Epub 1995/10/01. . 7557388 10.1101/gad.9.19.2364

[49] Sengupta S, Peterson TR, Laplante M, Oh S, Sabatini DM. mTORC1 controls fasting-induced ketogenesis and its modulation by ageing. Nature. 2010;468(7327):1100–4. 10.1038/nature09584 . 21179166

[50] Magee JA, Ikenoue T, Nakada D, Lee JY, Guan KL, Morrison SJ. Temporal changes in PTEN and mTORC2 regulation of hematopoietic stem cell self-renewal and leukemia suppression. Cell Stem Cell. 2012;11(3):415–28. Epub 2012/09/11. 10.1016/j.stem.2012.05.026 . 22958933 PMC3447536

[51] Prigge JR, Wiley JA, Talago EA, Young EM, Johns LL, Kundert JA, et al Nuclear double-fluorescent reporter for in vivo and ex vivo analyses of biological transitions in mouse nuclei. Mamm Genome. 2013 Epub 2013/09/12. 10.1007/s00335-013-9469-8 . 24022199 PMC3952041

[52] Soriano P. Generalized lacZ expression with the ROSA26 Cre reporter strain. Nat Genet. 1999;21(1):70–1. Epub 1999/01/23. 10.1038/5007 . 9916792

[53] Ishii M, Arias AC, Liu L, Chen YB, Bronner ME, Maxson RE. A stable cranial neural crest cell line from mouse. Stem Cells Dev. 2012;21(17):3069–80. 10.1089/scd.2012.0155 . 22889333 PMC3495126

