# Supplementary figures and images for: mTOR acts as a pivotal signaling hub for neural crest cells during craniofacial development
Source: PLoS Genet. 2018 Jul 5;14(7):e1007491. doi: 10.1371/journal.pgen.1007491 (PMC6049956; doi:10.1371/journal.pgen.1007491)

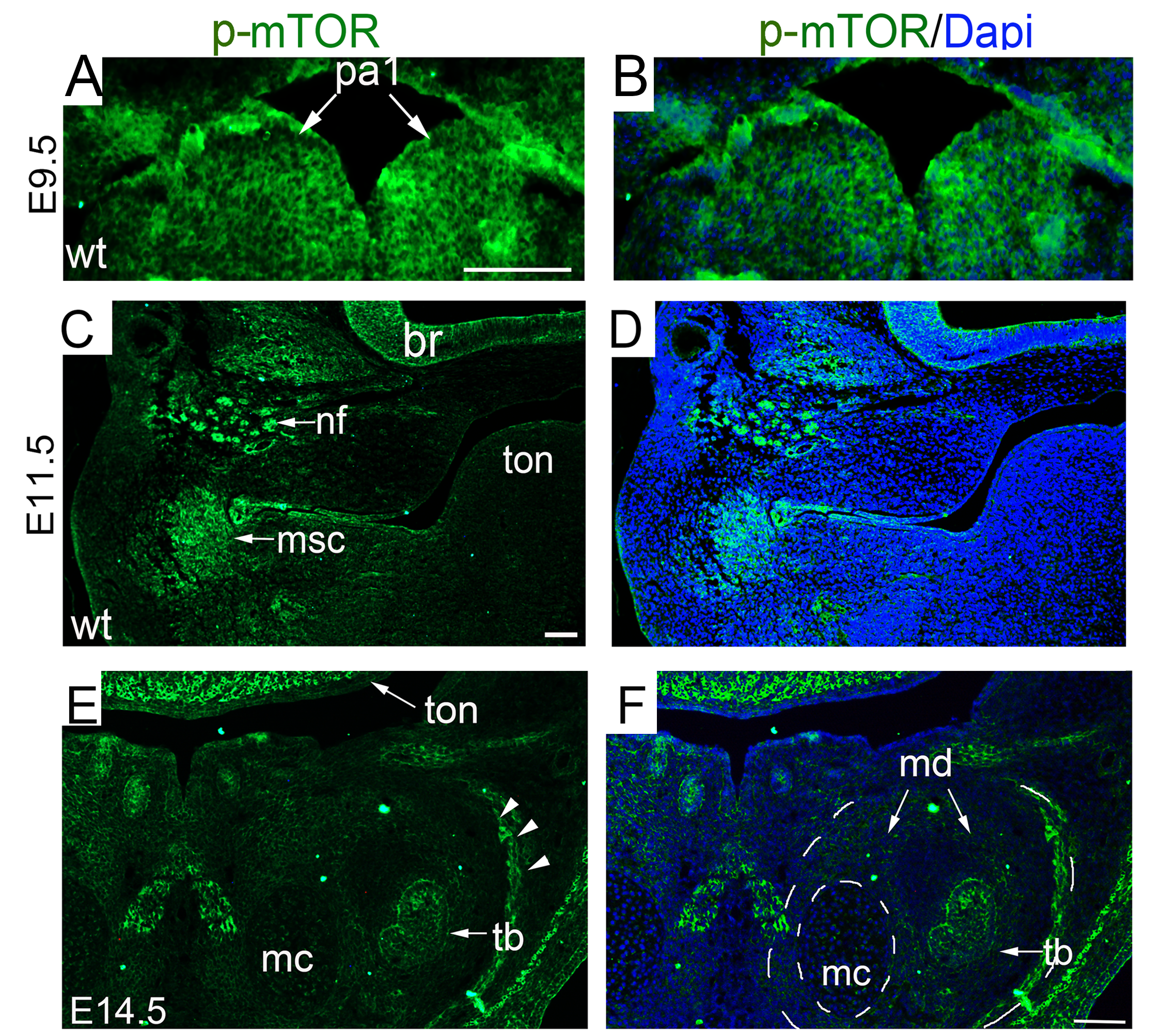

Supplement: S1 Fig — (A, B) Immunofluorescence for p-mTOR of the first PA at E9.5. (C, D) Immunofluorescence for p-mTOR at E11.5. (E, F) Immunofluorescence for p-mTOR at E14.5. br: brain; mc: Meckel’ s cartilage; md: mandible; msc: mesenchymal condensate; nf: neurofilament; pa: pharyngeal arch; tb: tooth bud; ton: tongue. Scale bar: 100 μm. (TIF) [file pgen.1007491.s001.tif]

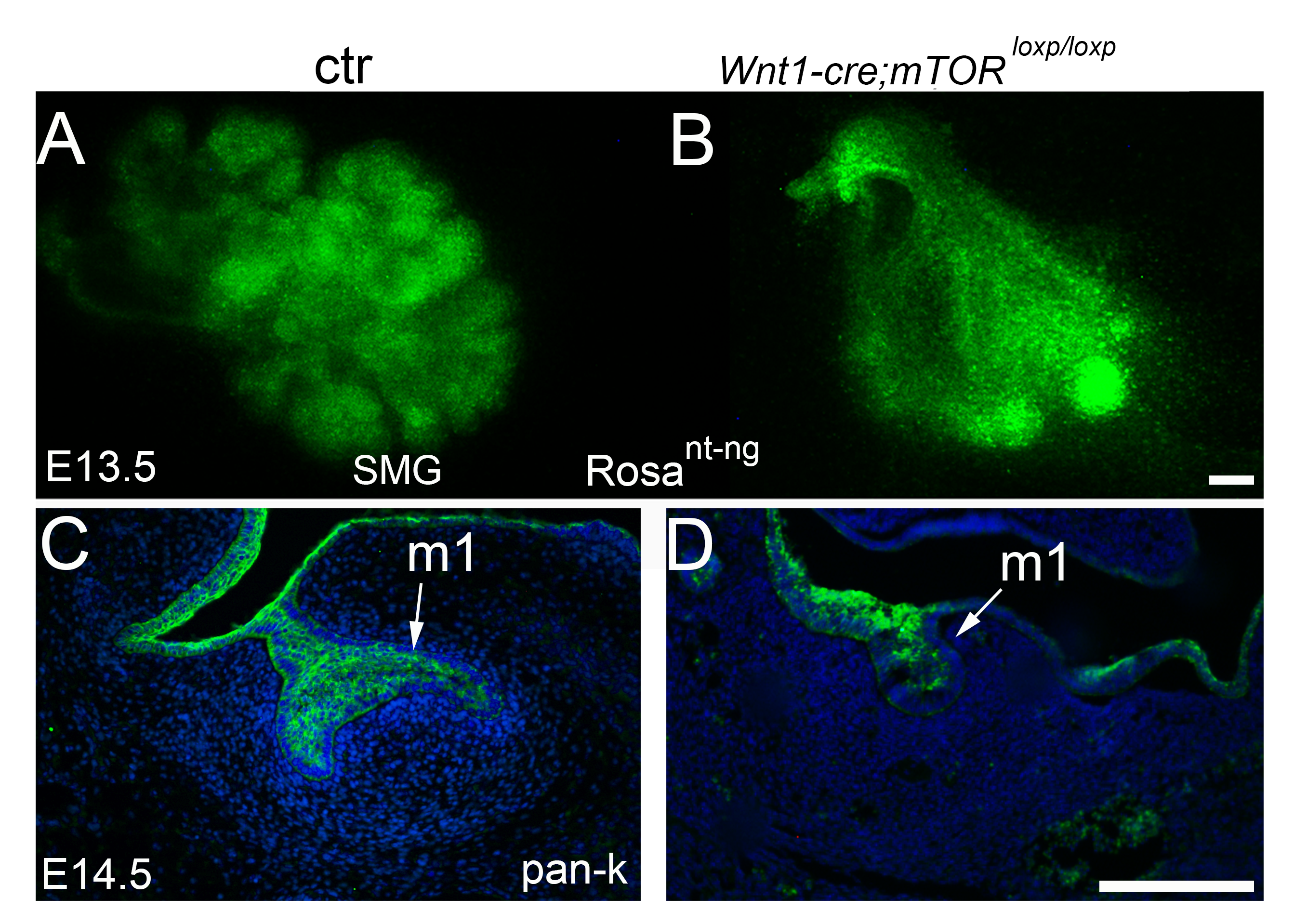

Supplement: S2 Fig — (A, B) Gross examination of the submandibular gland with a fluorescent microscope at E13.5. (C, D) Mandibular first molar stained with pan-keratin. Development of mutant molar is arrested at the bud stage. pan-k: pan-keratin; m1: the first molar; smg: submandibular gland. Scale bar: 100μm. (TIF) [file pgen.1007491.s002.tif]

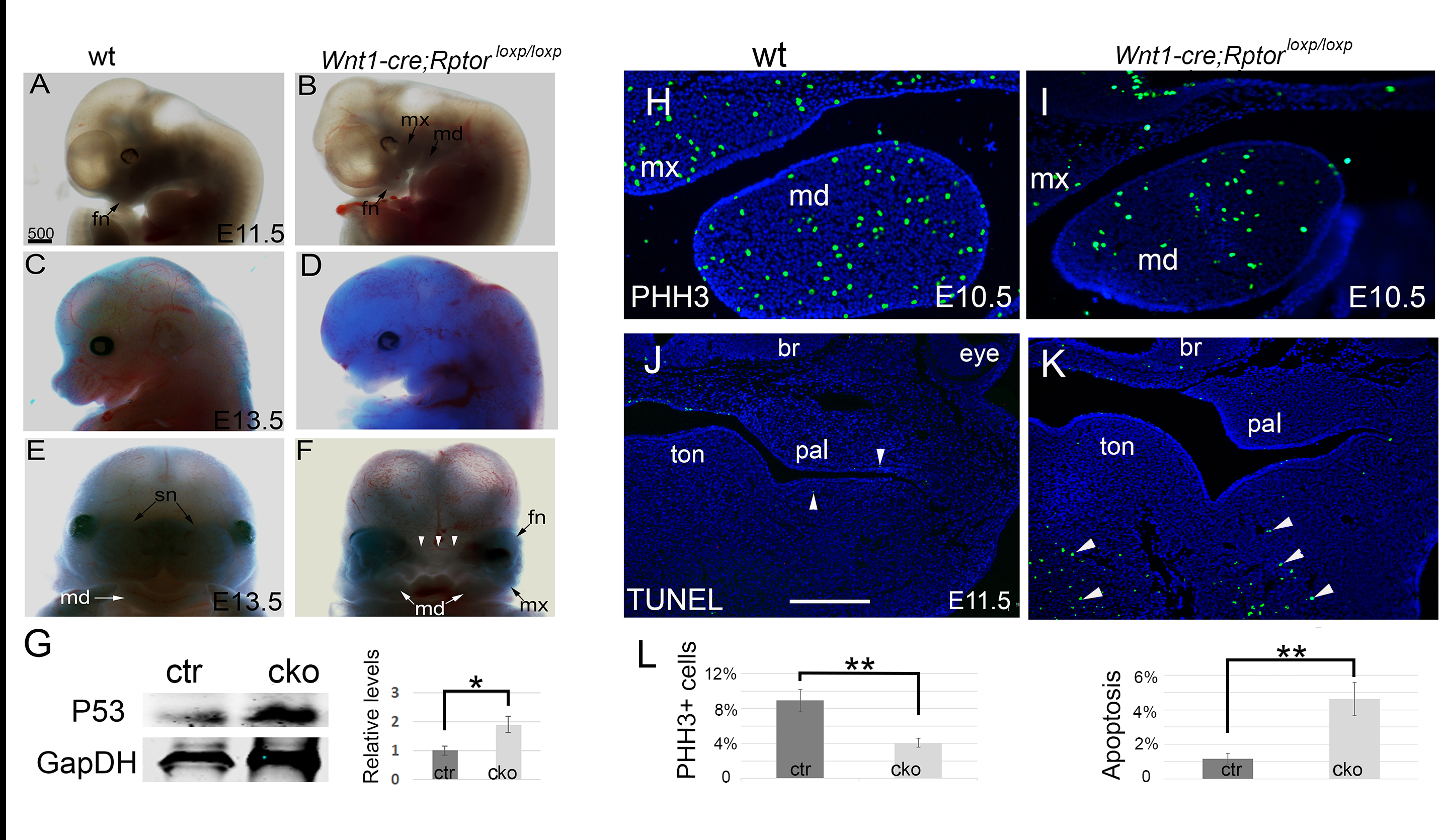

Supplement: S3 Fig — (A, B) Lateral view of E11.5 embryos. (C-F) Lateral and frontal view of E13.5 embryos. Arrowheads in (F) indicate midline cleft in the mutant. (G) Quantification of P53 levels of E11.5 mouse facial primordia, *P<0.05. (H, I) PHH3 staining at E10.5. (J, K) Apoptosis at E11.5. (L) Quantification of PHH3+ cells and apoptotic cells, **p<0.01. br: brain; fn: frontonasal prominence; md: mandibular prominence; mx: maxillary prominence: pal: palate; sn: snout; ton: tongue. Scale bar (A-F): 500 μm; (G-J): 100 μm. (TIF) [file pgen.1007491.s003.tif]
